# Surgical treatment of specific Unified Classification System B fractures: potentially destabilising lesser trochanter periprosthetic fractures

**DOI:** 10.1038/s41598-023-41698-x

**Published:** 2023-08-31

**Authors:** Wei-Qiang Zhao, Xu-Song Li, Meng-Qiang Fan, Zhi-Yuan Yao, Zhou-Feng Song, Pei-Jian Tong, Jie-Feng Huang

**Affiliations:** 1 Department of Orthopaedics and Traumatology, The First Affiliated Hospital of Zhejiang Chinese Medical University, 54 Youdian Road, Shangcheng District, Hangzhou, 310006 China; 2 The First Clinical College, Zhejiang Chinese Medical University, Hangzhou, 310006 Zhejiang China; 3 Department of Orthopaedics and Traumatology, Zhongshan Hospital of Traditional Chinese Medicine, Zhongshan, 528401 Guangdong China

**Keywords:** Diseases, Medical research

## Abstract

To investigate the clinical effects of specific Unified Classification System B (UCS B)-lesser trochanter periprosthetic fractures and determine whether they occur only with non-cemented stems. A retrospective analysis of 28 patients with specific UCS B2 fractures who underwent two surgical treatments, longer stem revision and internal fixation (LSRIF) and open reduction and internal fixation (ORIF), was performed. The patients were assessed at 1, 3, 6, 12, and 24 months and annually thereafter. Fracture healing, complications, Harris Hip Score (HHS), and the Short Form Health Survey questionnaire (SF-36) quality of life score were assessed at each follow-up. At the time of the last follow-up, seven patients had been lost: three were lost to contact, two died, and two were hospitalised elsewhere and unavailable for follow-up. The remaining 21 patients were followed for an average of 49.3 ± 15.4 (range: 24–74.4) months. Their average fracture healing time was 13.5 ± 1.1 (12–15.4) weeks. Complications included three cases (10.71%) of thrombus, one (3.57%) of heart failure, and one (3.57%) of pulmonary infection. There were no revisions due to prosthesis loosening, subsidence, or infection. At the last follow-up, the HHS, SF-36 mental score, and SF-36 physical score were recorded, LSRIF *vs.* ORIF (82.9 ± 6.6 *vs*. 74.7 ± 3.9, *p* = *0.059*; 50.9 ± 7.6 *vs*. 38 ± 1.4, *p* = *0.012*, and 51.7 ± 8.4 *vs*. 39.7 ± 3.4, *p* = *0.032*, respectively). Specific UCS B2 fractures mostly occur with non-cemented stems. LSRIF with cables is the main treatment, while ORIF is an option for those elderly in poor condition.

## Introduction

Periprosthetic femur fracture (PPFF) is an acute complication that often occurs after hip arthroplasty. Due to the increasing use of arthroplasty surgery and the aging population, the incidence of PPFF is increasing^1–4^.

The Vancouver Classification System (VCS)^5^ and subsequent Unified Classification System (UCS)^6^ were intended as simple systems for classifying fractures and guiding management. However, these two classifications do not include some special types of fracture. For example, Van Houwelingen and Duncan^7^ reported a type of periprosthetic fracture of the lesser trochanter that involved a segment of the proximal medial femoral cortex. This fracture is easily misidentified as an A_LT_ fracture, but is actually type B2. Consequently, it is called a “pseudo A_LT_” or “new B2” fracture. Pseudo A_LT_ fractures often occur in non-cemented stems^7–11^; the mechanism may involve an occult fracture resulting from surgical insertion, and the fractures occur during early weight-bearing rehabilitation.

To assess the importance to this type of fracture and improve the surgical outcome, we hypothesized pseudo A_LT_ fractures occur only with non-cemented stems and examined how different surgical treatments of pseudo A_LT_ fractures affect the clinical outcome.

## Materials and methods

This study was approved by the Hospital Ethics Committee. All methods were carried out in accordance with relevant guidelines and regulations. All X-rays were preoperatively evaluated by two senior doctors to confirm the type of fracture. Any disagreement between two doctors was resolved by a third doctor. The final determination of whether the prosthesis was loose was made during the operation.

### Inclusion and exclusion criteria

The inclusion criteria were confirmed pseudo A_LT_ fractures on X-ray, surgical treatment, and complete clinical records and follow-up. Exclusion criteria were a combined (or suspected) infection, ipsilateral lower-extremity neurovascular injury, conservative (non-surgical) treatment, and incomplete follow-up.

### Patient-related information

A search of the hospital database from January 2010 to July 2020 identified 28 patients. The patients’ basic data were collected, including gender, age, preoperative physical condition, surgical index, and type of stem.

### Treatment methods

**Figure 1:**
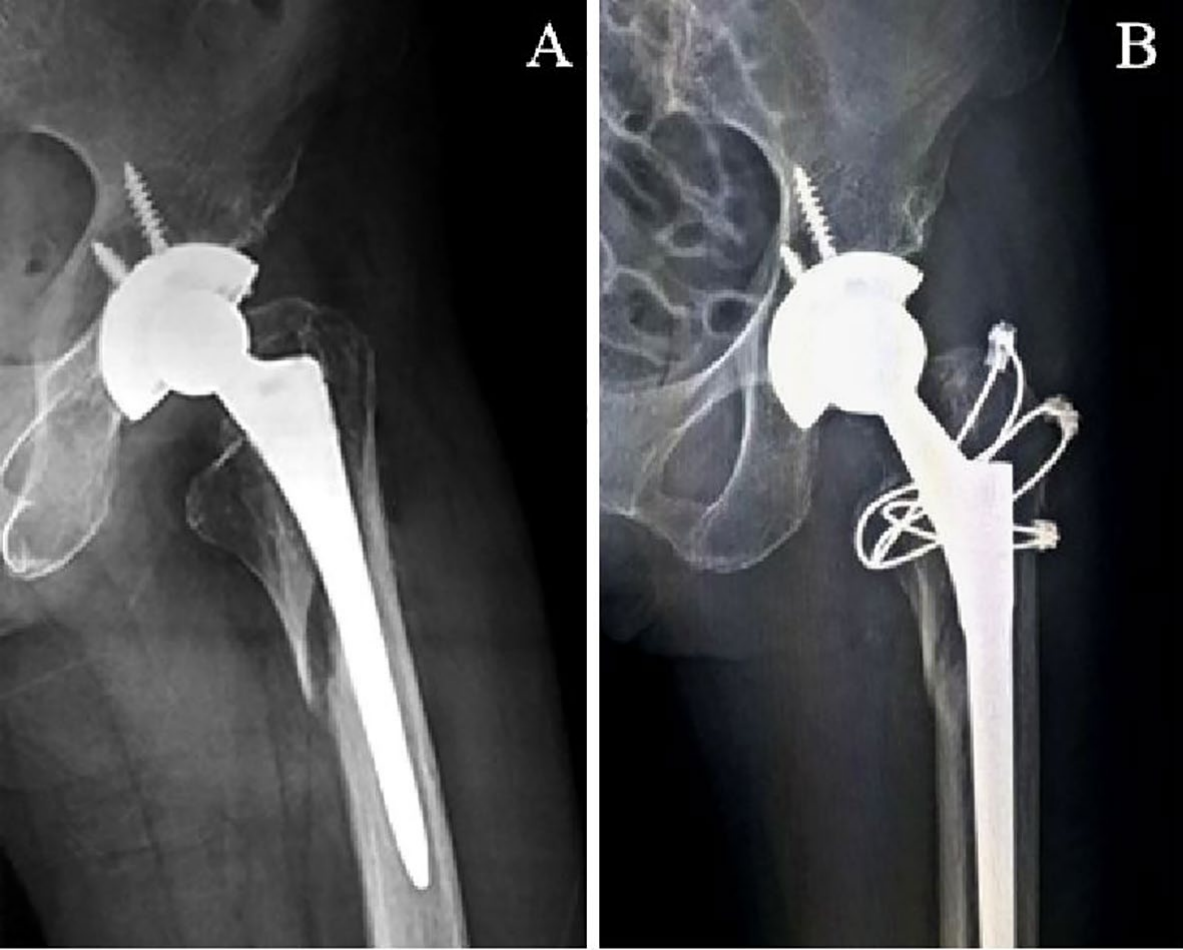
Case 2, a 60-year-old woman with a left femoral neck fracture due to a fall. (**A**) Preoperative lateral radiograph. (**B**) Postoperative anterior–posterior radiograph after longer stem revision and internal fixation with cables.

**Figure 2:**
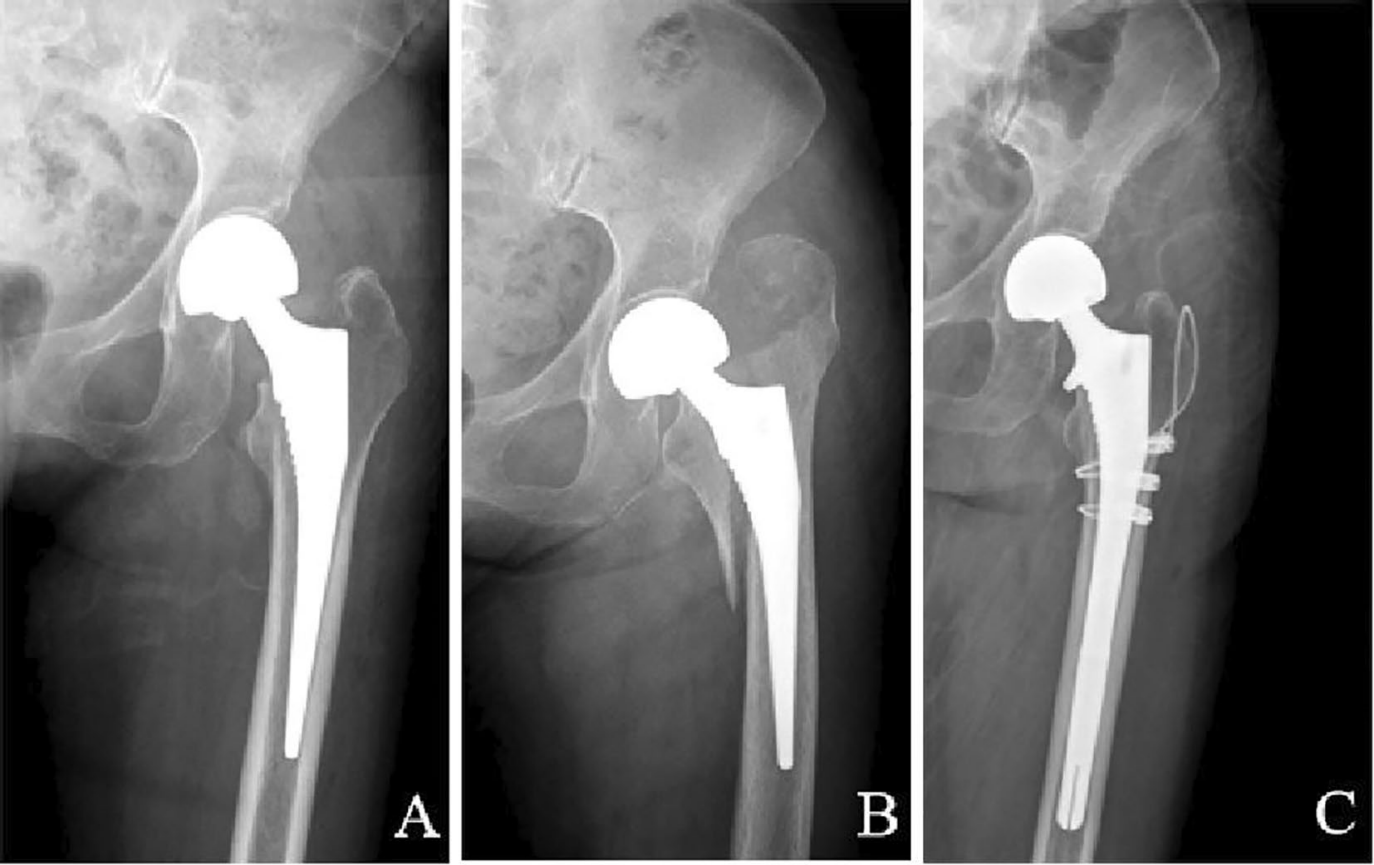
Case 8, an 81-year-old woman. (**A**) Anterior–posterior radiograph after hemi-arthroplasty. (**B**) After a fall in the ward, the continuity of the left medial femoral cortex was disrupted on anterior–posterior and lateral radiographs. (**C**) Postoperative anterior–posterior radiograph shows the patient with a longer stem revision and internal fixation with cables.

**Figure 3:**
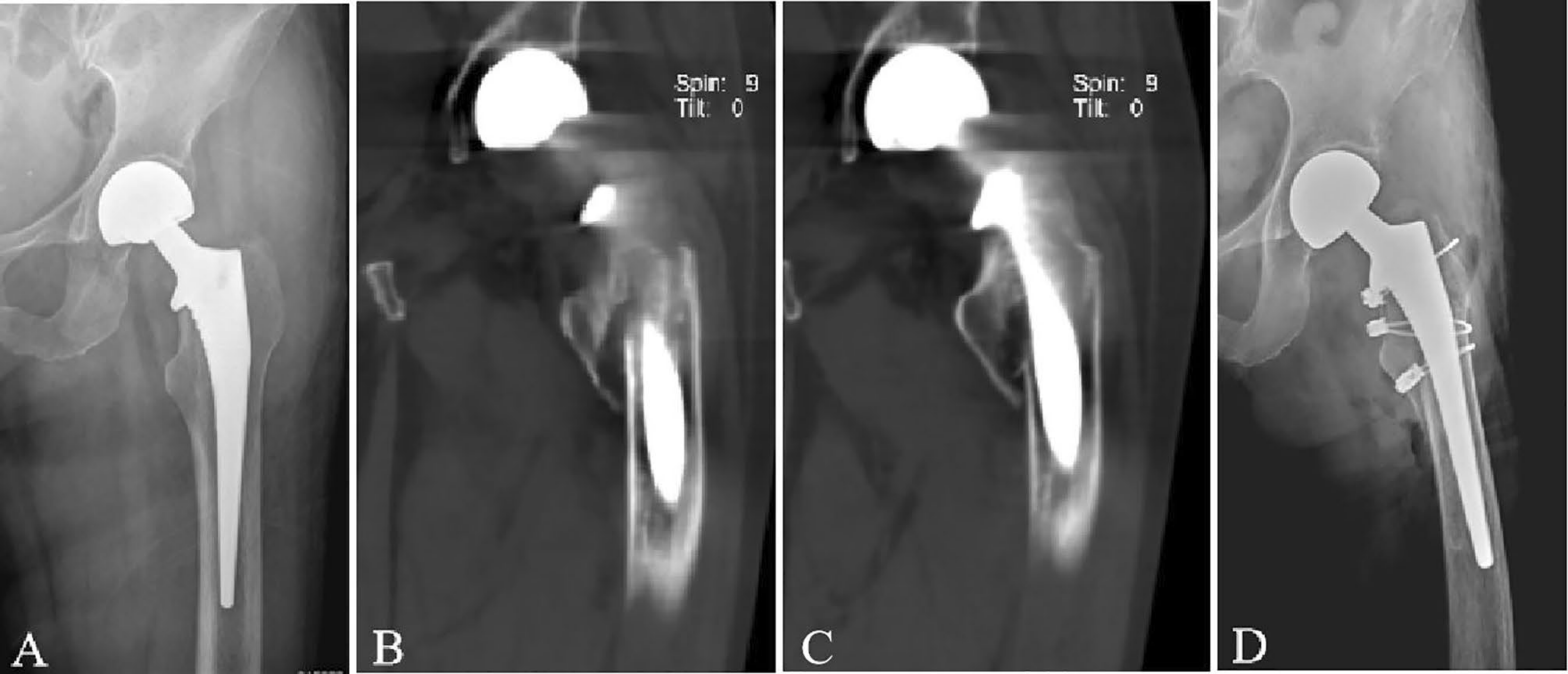
Case 19, an 88-year-old woman. (**A**) Anterior–posterior radiograph after hemi-arthroplasty. (**B–C**) Two coronal CT images show the periprosthetic fracture. (**D**) Anterior–posterior radiograph shows the surgical treatment of open reduction and internal fixation with cables.

Two treatment methods were adopted. Longer stem revision and internal fixation (LSRIF) with cables was applied in 24 cases (Figs. 1 and 2). Open reduction and internal fixation (ORIF) with cables was done in four cases (Fig. 3). The choice of surgical treatment was based on the patient’s physical condition. The standard treatment was LSRIF, while ORIF was chosen if the patient was in poor condition and could not tolerate LSRIF.

Anticoagulant therapy with low-molecular-weight heparin was given postoperatively. Rehabilitation was based on the patient’s physical condition. Patients were allowed early weight bearing when the circumstances permitted; otherwise, they exercised in bed.

### Follow-up

All patients were followed as outpatients at 1, 3, 6, 12, and 24 months and yearly thereafter. All surviving patients with complete follow-up were analysed. Fracture healing and the position of the prosthesis loosening were evaluated by X-rays at each visit. The Harris Hip Score (HHS)^12^ and Short Form Health Survey questionnaire (SF-36) quality of life score^13^ were also recorded. Fracture healing was assessed in terms of local tenderness, longitudinal percussion pain on the injured leg, local abnormalities, and blurred fracture lines with continuous callus passing through the fracture line on X-ray at the last follow-up. The fracture healing time was defined as the time from the second postoperative day to the end of follow-up if there were no special circumstances.

### Statistical analysis

The data were analysed using SPSS software (ver. 22.0; SPSS Inc., Chicago, IL, USA). Data are expressed as mean ± standard deviation (SD). The groups were compared using *t*-tests. Count data are expressed as numbers or percentages. *P*-values < 0.05 were considered significant.

### Ethical approval

Approved by the Ethics Committee of the First Affiliated Hospital of Zhejiang Chinese Medicine University, Ethics No. 2016-K-143-01.

### Informed consent

Informed consent was obtained from all participants.

## Results

**Figure 4:**
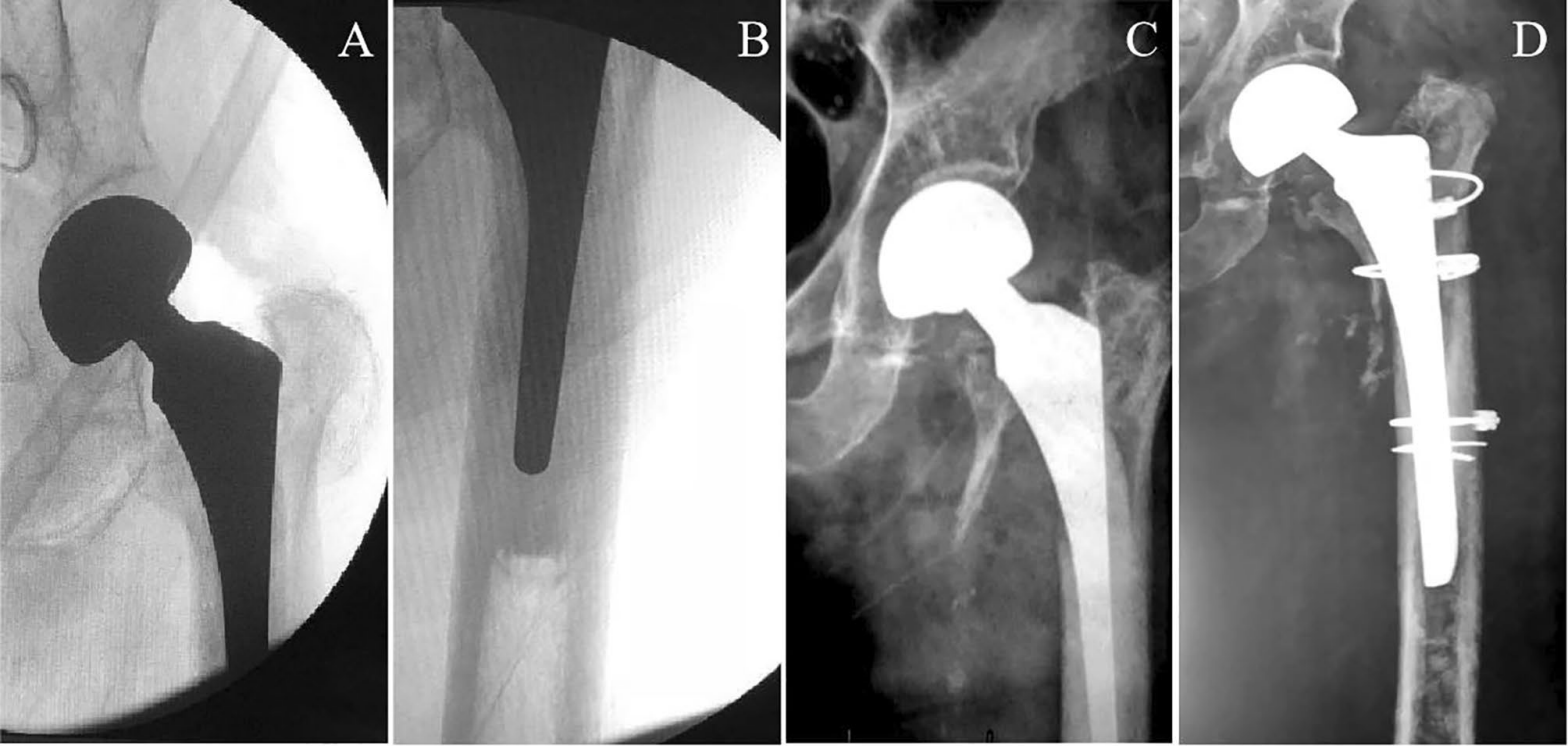
Case 1, an 82-year-old man underwent left hemi-arthroplasty with a cemented stem because of a left femoral neck fracture. (**A–B**) Two intraoperative radiographs taken with a C-arm machine. (**C**) After a fall, the continuity of the left medial femoral cortex was interrupted on an anterior–posterior radiograph. (**D**) Postoperative anterior–posterior radiograph after longer stem revision and internal fixation with cables.

Postoperative pseudo A_LT_ fractures were seen in 28 patients (7 males [25% and 21 females [75%]). The age of the patients at surgery was 73.7 (range: 52–92) years. Of the cases, 27 (96.43%) occurred with non-cemented stems, and 1 (3.57%) with cemented stems (Fig. 4). The basic patient data are summarised in Table 1.

**Table 1:** Basic patient data.

Periprosthetic femur fracture new B2	
Number of hips	28
Age at surgery, mean (range)	73.7 (52–92) years
Male: female ratio	7:21
Non-cemented stems: cemented stems ratio	27:1
LSRIF (%): ORIF (%) treatment ratio	24 (85.71%): 4 (14.29%)

LSRIF, longer stem revision and internal fixation with cables; ORIF, open reduction and internal fixation with cables.

At the time of the last follow-up, three patients were lost to contact, two died, and two were hospitalised elsewhere. The remaining 21 patients were followed for 49.3 ± 15.4 (24–74.4) months. These patients had a fracture healing time of 13.5 ± 1.1 (12–15.4) weeks.

Postoperative complications included thrombus in three cases (10.71%) and heart failure and pulmonary infection in one case each (both 3.57%). The patients with postoperative thrombosis all recovered on treatment with low-molecular-weight heparin. There were no revisions for prosthesis loosening, subsidence, or infection. The HHS and SF-36 scores were obtained scores those with complete follow-up (Table 2).

**Table 2:** Patient demographic and medical data.

Number	Age (years)	Gender	Type of operation	Type of stem	Follow-up (months)	Complications	HHS	SF-36	
								Mental	Physical
1	86	M	LSRIF	Cemented	50.4	No	68	38	36
2	60	F	LSRIF	Non-cemented	61.2	No	88	60	60
3	59	M	LSRIF	Non-cemented	36	No	92	62	65
4	86	M	LSRIF	Non-cemented	50.4	No	68	38	36
5	77	F	LSRIF	Non-cemented	70.8	No	82	47	50
6	62	F	LSRIF	Non-cemented	25.2	Heart failure	87	55	52
7	71	F	LSRIF	Non-cemented	44.4	No	84	52	55
8	81	F	LSRIF	Non-cemented	42	No	81	42	49
9	84	F	LSRIF	Non-cemented	74.4	No	82	49	48
10	75	F	LSRIF	Non-cemented	57.6	No	84	49	41
11	76	F	LSRIF	Non-cemented	37.2	Thrombus	86	50	51
12	61	M	LSRIF	Non-cemented	67.2	NO	87	56	58
13	85	F	LSRIF	Non-cemented	46.8	No	74	42	47
14	72	M	LSRIF	Non-cemented	52.8	Thrombus	83	52	51
15	72	F	LSRIF	Non-cemented	28.8	No	85	51	52
16	52	F	LSRIF	Non-cemented	26.4	Thrombus	90	64	67
17	54	F	LSRIF	Non-cemented	73.2	No	89	61	61
18	79	F	LSRIF	Non-cemented	24	No	82	48	51
19	88	F	ORIF	Non-cemented	63.6	Pulmonary infection	73	37	35
20	85	F	ORIF	Non-cemented	49.2	No	80	40	41
21	91	M	ORIF	Non-cemented	54	No	71	37	43
22	64	M	LSRIF	Non-cemented	21.6 (lost to follow-up)	No	–	–	–
23	61	F	LSRIF	Non-cemented	14.4 (hospitalised)	No	–	–	–
24	71	F	LSRIF	Non-cemented	10.8 (died)	No	–	–	–
25	87	F	LSRIF	Non-cemented	9.6 (lost to follow-up)	No	–	–	–
26	72	F	LSRIF	Non-cemented	15.6 (lost to follow-up)	No	–	–	–
27	60	F	LSRIF	Non-cemented	18 (hospitalised)	No	–	–	–
28	92	F	ORIF	Non-cemented	16.8 (died)	No	–	–	–

PPFF, periprosthetic femur fracture; M, male; F, female; HHS, Harris Hip Score; ORIF, open reduction and internal fixation with cables; LSRIF, longer stem revision and internal fixation with cables.

Of the 21 patients who completed follow-up, 18 (85.71%) underwent LSRIF and 3 (14.29%) underwent ORIF. At the last follow-up, the LSRIF and ORIF groups had an HHS of 82.9 ± 6.6 (range: 68–92) and 74.7 ± 3.9 (range: 71–80) (*p* = *0.059*), SF-36 mental scores of 50.9 ± 7.6 (range: 38–64) and 38 ± 1.4 (range: 37–40) (*p* = 0.012), and SF-36-physical scores of 51.7 ± 8.4 (range: 36–67) and 39.7 ± 3.4 (range: 35–43), respectively (*p* = *0.032*) (Table 3).

**Table 3:** Comparison of the characteristics and outcomes of the two groups.

Treatment methods	LSRIF	ORIF
Total hips (no.)	18	3
Gender (F:M)	14:4	2:1
Age (years)	71.8 ± 10.9 (52–86)	88 ± 2.4 (85–91)
Follow-up time (months)	48.3 ± 16.3 (24–74.4)	55.6 ± 6.0 (49.2–63.6)
Healing time (weeks)	13.4 ± 1.1 (12–15.4)	14.0 ± 0.8 (13.1–15)
Complications		
Thrombus (%)	3 (16.7%)	0
Heart failure	1 (5.6%)	0
Pulmonary infection	0	1 (33.3%)
HHS (range)	82.9 ± 6.6 (68–92)	74.7 ± 3.9 (71–80)
SF-36-mental (range)	50.9 ± 7.6 (38–64)	38 ± 1.4 (37–40)
SF-36-physical (range)	51.7 ± 8.4 (36–67)	39.7 ± 3.4 (35–43)

LSRIF, longer stem revision and internal fixation with cables; ORIF, open reduction and internal fixation with cables; HHS, Harris Hip Score; SF-36, Short Form Health Survey questionnaire quality of life score (including mental and physical).

## Discussion

The VCS divides fractures into three subtypes. Type A consists of fractures in the greater (A_GT_) and lesser (A_LT_) trochanteric areas. Type B fractures involve the metaphyseal or diaphyseal femur around the prosthetic stem: type B1 fractures occur around a stable stem; B2 fractures involve a loose stem with good bone quality; and B3 fractures involve bone loss. Type C fractures occur distal to the stem.

The UCS expanded and updated the VCS by adding three types. Type D is a femoral fracture occurring after hip or knee arthroplasty. Type E is a fracture of both the acetabulum and femur after hip arthroplasty. Type F is a fracture of the acetabulum after hemi-arthroplasty of the hip^4,6^ (Table 4).

**Table 4:** Summary of the Unified Classification System.

Type	Subtype	Fracture description
Type A		Fracture in the trochanteric area
	A_GT_	Fracture of the greater trochanter
	A_LT_	Fracture of the lesser trochanter
Type B		Fracture around or just below the stem
	B1	Well-fixed stem without loosening
	B2	Loose stem with good bone reserve
	B3	Loose stem with poor-quality bone
Type C		Fracture distal to the stem
Type D		Fracture of the femoral after hip and knee arthroplasty
Type E		Both the femur and acetabulum fractures after THA
Type F		Fracture of the acetabulum after hemi-arthroplasty of the hip

THA, total hip arthroplasty.

However, some special fractures are not included. For example, a pseudo A_LT_/new B2 fracture occurs at the lesser trochanter extending into the medial cortex of the proximal femur^7,8, 14–18^ (Table 5). Consequently, modified classifications based on the VCS and UCS were proposed. Capello et al. proposed “A1 and A2” for well-fixed or loose stems in category A^8^. Huang et al. classified type B2 as B2PAGT and B2PALT, representing greater trochanter fractures including a segment of the proximal lateral femoral cortex and lesser trochanter fractures including a segment of the proximal medial femoral cortex, respectively^15^. Egrise et al. subdivided Vancouver B-lesser trochanter fractures (VB-LT) into stable (VB-LT1) and unstable (VB-LT2) fractures^9^. These are all actually B2/pseudo A_LT_ fractures.

**Table 5:** Related reports on pseudo A_LT_ fractures.

Authors	Year	Mechanism of occurrence	Treatment
Van Houwelingen and Duncan^7^	2011	Non-cemented stem displaced under load with an unrecognised intraoperative fracture; may occur during rehabilitation	Longer stem revision and cerclage cable fixation
Capello et al.^8^	2014	An unrecognised intraoperative fracture Geometry of the tapered stem	6 treated with ORIF; 3 treated without surgery
Huang et al.^15^	2018	Proximal loosening of femoral prosthesis and poor-quality bone bed	Longer stem revision and internal fixation
Karam et al.^16^	2020	Anatomical and wedge design non-cemented stems	Revision and cerclage wire fixation
González-Martín, et al.^17^	2022	–	Longer stem revision and internal fixation
Egrise et al.^9^	2022	An occult intraoperative fracture	9 VB-LT1 (without subsidence) and 6 VB-LT2 in poor health treated nonoperatively; 13 VB-LT2 (with subsidence) with stem exchange and cerclage; 3 with isolated stem exchange; 2 with internal fixation by cerclage

ORIF, open reduction and internal fixation.

Pseudo A_LT_ fractures often occur with non-cemented stems, especially tapered stems^7,10,11^. This may be due to an unrecognised intraoperative fracture that is subsequently displaced under load or during rehabilitation^7–9,15, 16^. Capello et al. reported nine newly described “clamshell” fractures that might be related to unrecognised fractures during operation and believed that they were directly related to the geometry of the tapered stem used^8^. Huang et al. mentioned that this type of fracture occurred at the lesser trochanter, including a segment of the proximal medial femoral cortex, and was associated with destabilisation of the stem^15^. Karam et al. showed that such fractures were significantly associated with non-cemented supports with an anatomical wedge-shaped design^16^. Egrise et al. thought this type of fracture can occur intraoperatively or in the early postoperative period with non-cemented implants, and is an occult intraoperative fracture^9^.

In our series, 27 patients with non-cemented stems (96.43%) sustained a PPFF. The mechanism was as described above. One fracture involved a cemented stem (3.57%); this patient had osteoporosis and it might have been an occult fracture that was not found intraoperatively.

There are three common treatments for new B2 fractures in PPFF: LSRIF, ORIF, and conservative treatment. When Van Houwelingen and Duncan recognised an undisplaced cortical crack, they performed cerclage cable fixation and revision with a longer stem, with protected weight-bearing for 6 weeks^7^. Capello et al. reported nine cases of pseudo A_LT_ fracture caused by non-cemented femoral stems. They treated three cases conservatively without surgery because the stem subsidence stabilised during rehabilitation; the remaining six were treated by removing the loose stems and fixing the fracture and all healed well^8^; the remaining six were treated by removing the loose stems and fixing the fracture and all healed well^8^. Karam et al. adopted wedge-shaped (10 cases) and straight (20 cases) fixation treatments^16^. Egrise et al. treated nine VB-LT1 and six VB-LT2 in patients in poor health nonoperatively, 13 VB-T2 stems with exchange and cerclage, three with isolated stem exchange, and two with internal fixation by cerclage^9^. Huang et al.^15^ and González-Martín et al.^17^ treated this type with LSRIF.

In this study, of the 21 patients with complete follow-up, 18 underwent LSRIF and ORIF was used in 3 cases. The patient outcomes were determined by postoperative fracture-healing and functional assessment scores (HHS and SF-36). LSRIF had the better curative effect, not only in terms of the above but also from the average healing time of 13.4 ± 1.1 weeks. The LSRIF patients were aged 71.8 ± 10.9 (52–86) years and were younger than the ORIF patients (88 [range: 85–91] years). The mental and physical SF-36 scores were better with LSRIF (50.9 ± 7.6 and 51.7 ± 8.4, respectively) than with ORIF (38 ± 1.4 and 39.7 ± 3.4, respectively); we attributed this to the patients’ age and clinical condition. The HHS exceed 70 in all patients except case 1 (Table 2), indicating good functional recovery after surgery. The HHS of case 1 was 68, which we attributed to the patient’s poor basic condition and severe osteoporosis. Moreover, revision surgery with a cemented stem was more difficult than conventional LSRIF. Compared with LSRIF, ORIF is less invasive and is a suitable choice for the elderly with poor physical condition and low living needs^3^. Smitham et al. confirmed that the anatomical reduction and ORIF of type B2 fractures should be considered an appropriate treatment for frail elderly patients with a PPFF around cemented polished double-tapered stems^14^.

This study has some limitations. The patients were from a single hospital, limiting the generalisability of the results. Given the retrospective nature of the study, multicentre prospective research is needed.

## Conclusion

Pseudo A_LT_ fractures result mainly from unrecognised occult fractures intraoperatively and improper exercise postoperatively. They involve a posteromedial cortical fragment around the lesser trochanter and mainly occur with non-cemented stems. While the main treatment is LSRIF with cables/plates, simple ORIF can achieve good healing, especially for elderly patients in poor general condition.

## Data Availability

The datasets used and/or analysed during the current study available from the corresponding author on reasonable request.
